# Supplementary material for: Multi-chiral materials comprising metallosupramolecular and covalent helical polymers containing five axial motifs within a helix
Source: Nat Commun. 2023 Jun 8;14:3348. doi: 10.1038/s41467-023-39014-2 (PMC10250298; doi:10.1038/s41467-023-39014-2)
Supplement: Supplementary file 1 — Supplementary information [file 41467_2023_39014_MOESM1_ESM.pdf]

Supplementary Information

Multi-Chiral Materials comprising Metallosupramolecular and Covalent Helical Polymers containing Five Axial Motifs within a Helix

Francisco Rey-Tarrío, Emilio Quiñoá, Gustavo Fernández and Félix Freire

Contents

Supplementary Methods……………………………………………………………...S1

Structures of the synthesized compounds…………………………………………S2

Synthesis of Ligands………………………………………………………………S3

General protocol for complexation and deprotection of monomers………………S6

Synthesis of Polymers……………………………………………………………. S9

Elemental Analysis…………………………………………………………….. S11

Spectroscopy Studies…………………………………………………………... S12

Thermal Studies………………………………………………………………... S13

VT-CD Experiments…………………………………………………………… S14

Photoluminescence properties and IR spectra………………………………… S15

Linear Dichroism Experiments (LD)…………………………………………....S16

Computational Details……………………………………………………………S19

Polymerization Degree effects…………………………………………………. S20

Supplementary References……………………………………………………….S21

Supplementary Methods

Reactions were conducted in dry solvents under argon unless otherwise stated. Et_3N was freshly distilled from CaH_2 under argon atmosphere. $[Rh(nbd)Cl]_2$ was obtained from Sigma-Aldrich (96%). All other chemicals were purchased from Sigma-Aldrich, Acros Organics, Alfa Aesar, Fluorochem, TCI Chemicals or Abcr and they were used as received.

Reaction mixtures were stirred using Teflon-coated magnetic stir bars. Thin layer chromatography (TLC) was carried out on pre-coated silica gel F254 plates with visualization under UV light or by dipping the plate into solutions of phosphomolybdic acid or potassium permanganate solutions followed by heating. Column chromatography was performed on silica gel (40-60 μm) unless otherwise stated.

CD measurements were performed on a Jasco-720, VT-CD and LD measurements on a Jasco-1500 with a 1 mm quartz cuvette. The amount of polymer used for CD, LD and VT-CD measurements was 0.6 mg/mL.

UV spectra were registered on a Jasco V-750 with a 1 mm quartz cuvette. The amount of polymer used for UV measurements was 0.6 mg/mL.

Optical rotation was measured on a Jasco-P2000.

Mass spectra were obtained on an HP 5988A spectrometer (Hewlett-Packard, Palo Alto, CA, USA).

Elemental analyses were recovered in a Foss-Heraeus CHNO-Rapid analyzer.

NMR experiments were measured on a Varian 300 (^1H: 300 MHz) or a Bruker Avance II 400 (^1H: 400 MHz; ^{13}C: 101 MHz; ^{19}F: 376.4 MHz). Solid NMR experiments were measured on a Bruker NEO 750 using a 1.3 mm rotor.

Raman spectra were performed on a Renishaw confocal Raman spectrometer (Invia Reflex model), equipped with two lasers (diode laser 785 nm and Ar laser 514 nm).

DSC traces were obtained on a DSC Q200 Tzero Technology (TA Instruments, New Castle, UK), equipped with a refrigerated cooling system RCS90 (TA Instruments, New Castle, UK), using a Tzero low-mass aluminium pan.

TGA traces were obtained on a TGA Q5000 (TA Instruments, New Castle, UK) using a platinum pan.

Photoluminescence spectra were obtained on a Horiba Fluoromax-Plus-C fluorimeter using a 10 mm quartz cuvette.

IR spectra were obtained on a JASCO-FTIR-6800. IR spectra in solution were recorded using a CaF2 cell with a path length of 0.1 mm.

Structures of the synthesized compounds

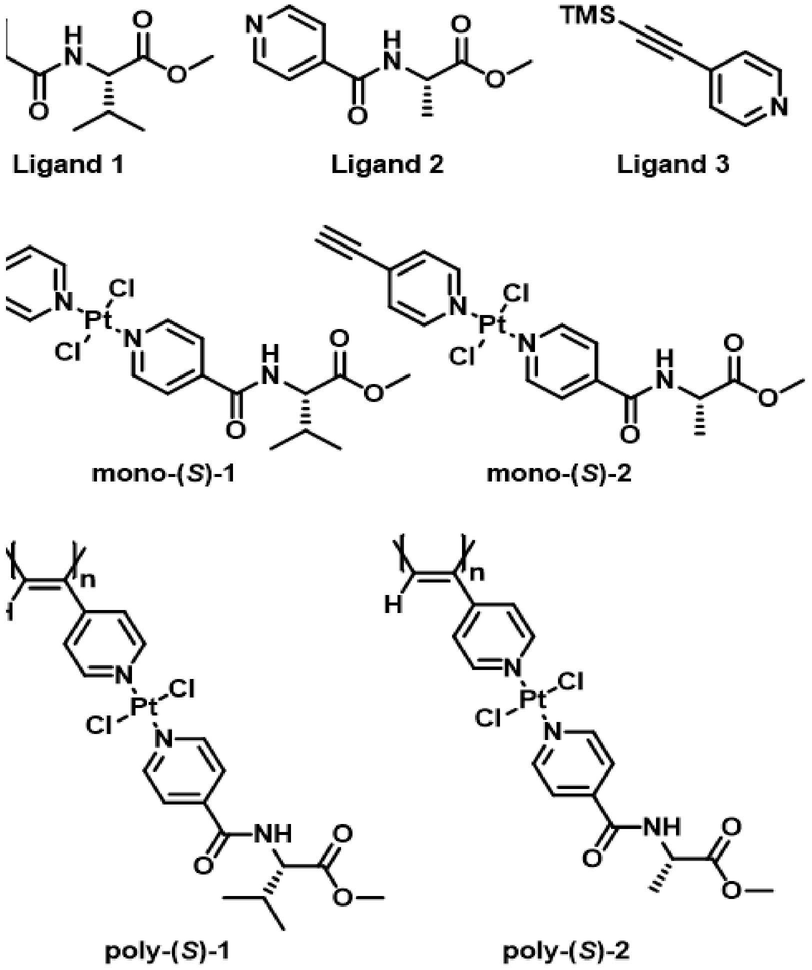

Supplementary Figure 1. Structures of the synthesized compounds.

Synthesis of Ligands

Methyl isonicotinoyl-*L*-valinate [Ligand 1]

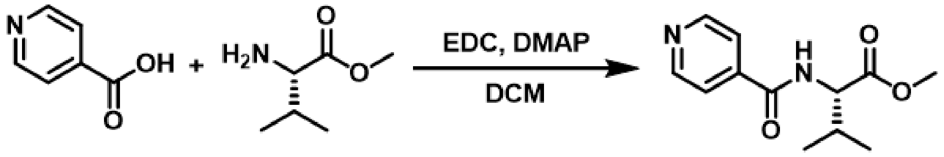

Isonicotinic acid (441 mg, 1.20 equiv.), 1-ethyl-3-(3-dimethylaminopropyl)carbodiimide (EDC, 686 mg, 1.20 equiv.), 1-hydroxybenzotriazole (HOBt, 484 mg, 1.20 equiv.) and 4-dimethylaminopyridine (DMAP, 437 mg, 1.20 equiv.) were dissolved in 30 mL of dry CH_2Cl_2. After 15 minutes, time needed to activate the acid, *L*-valine methyl ester (500 mg, 1.00 equiv.) was added and the mixture was stirred overnight. The organic layer was washed three times with HCl 1M and a saturated solution of $NaHCO_3$. The combined organic layers were dried over anhydrous Na_2SO_4, filtered and evaporated at reduced pressure. The crude product was chromatographed on silica gel with pentane/ethyl acetate (70:30) as eluent (388 mg, 55 % of yield).

^{1}H NMR (400 MHz, $CDCl_3$) δ_H (ppm): 0.89 (t, 6H), 2.16 (sextet, 1H), 3.67 (s, 1H), 4.64 (q, 1H), 7.21 (d, 1H), 7.54 (d, 2H), 8.59 (d, 2H).

^{13}C NMR (101 MHz, $CDCl_3$) δ_c (ppm): 18.1, 19.0, 31.3, 52.3, 57.7, 121.1, 141.2, 150.4, 165.6, 172.3.

HRMS (ESI-MS) m/z calcd for $C_{12}H_{16}N_2O_3Na^+$ $[M+Na]^+$: 259.1053, found: 259.1051

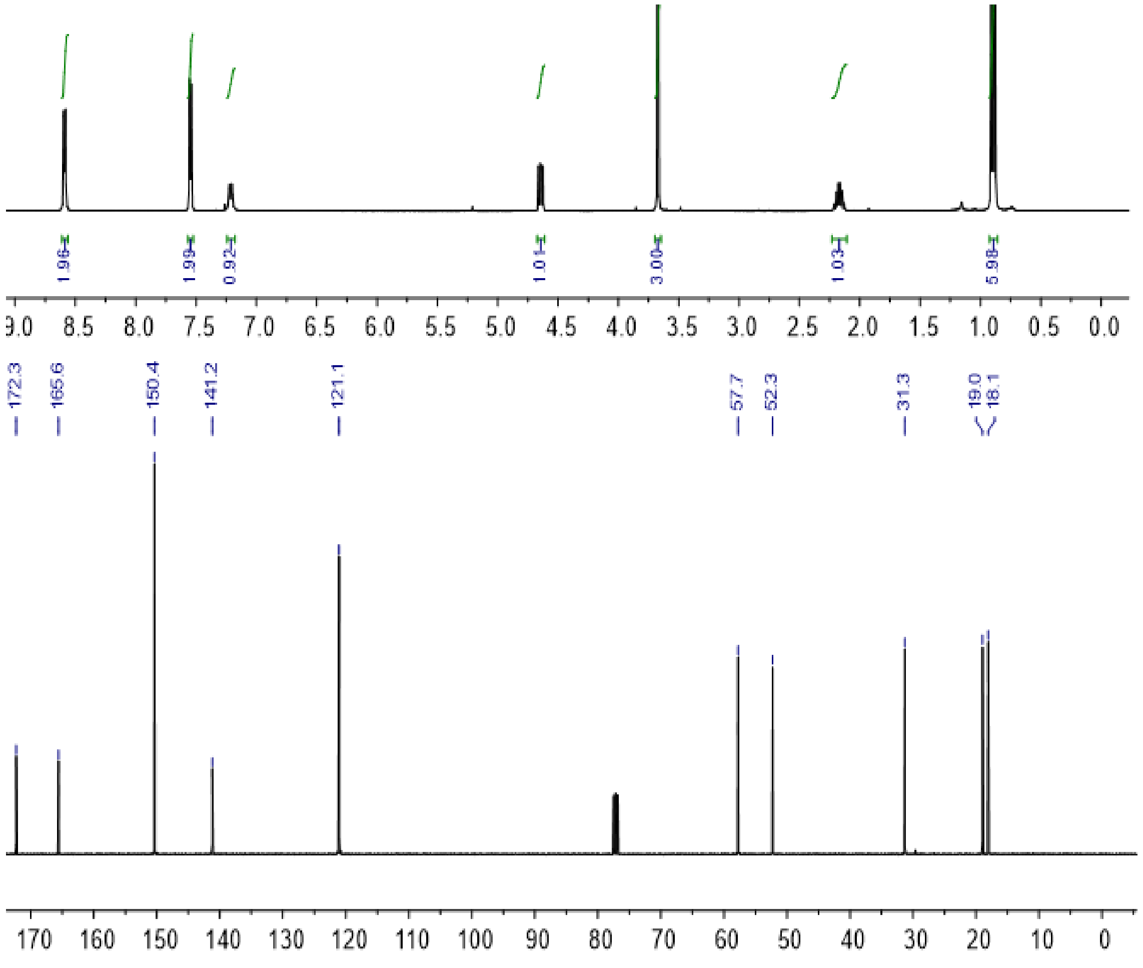

Supplementary Figure 2. ^{1}H and ^{13}C NMR spectra of ligand **1** ($CDCl_3$, 400 and 101 MHz respectively).

Methyl isonicotinoyl-*L*-alaninate [Ligand 2]

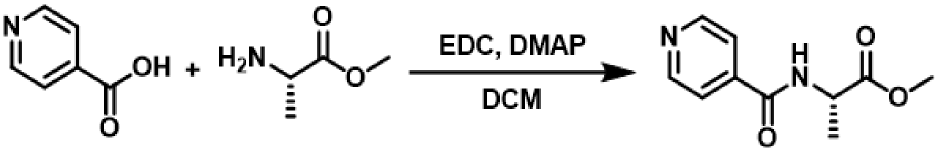

Isonicotinic acid (529 mg, 1.20 equiv.), 1-ethyl-3-(3-dimethylaminopropyl)carbodiimide (EDC, 824 mg, 1.20 equiv.), 1-hydroxybenzotriazole (HOBt, 581 mg, 1.20 equiv.) and 4-dimethylaminopyridine (DMAP, 525 mg, 1.20 equiv.) were dissolved in 30 mL of dry CH_2Cl_2. After 15 minutes, time needed to activate the acid, *L*-alanine methyl ester (500 mg, 1.00 equiv.) was added and the mixture was stirred overnight. The organic layer was washed three times with HCl 1M and a saturated solution of $NaHCO_3$. The combined organic layers were dried over anhydrous Na_2SO_4, filtered and evaporated at reduced pressure. The crude product was chromatographed on silica gel with pentane/ethyl acetate (70:30) as eluent (395 mg, 53 % of yield).

^{1}H NMR (400 MHz, $CDCl_3$) δ_H (ppm): 1.51 (d, 3H), 3.79 (s, 1H), 4.79 (quint, 1H), 7.52 (d, 1H), 7.66 (d, 2H), 8.68 (d, 2H).

^{13}C NMR (101 MHz, $CDCl_3$) δ_c (ppm): 18.3, 48.9, 52.9, 121.4, 141.3, 150.6, 165.4, 173.6.

HRMS (ESI-MS) m/z calcd for $C_{10}H_{12}N_2O_3Na^+$ $[M+Na]^+$: 231.0740, found: 231.0735.

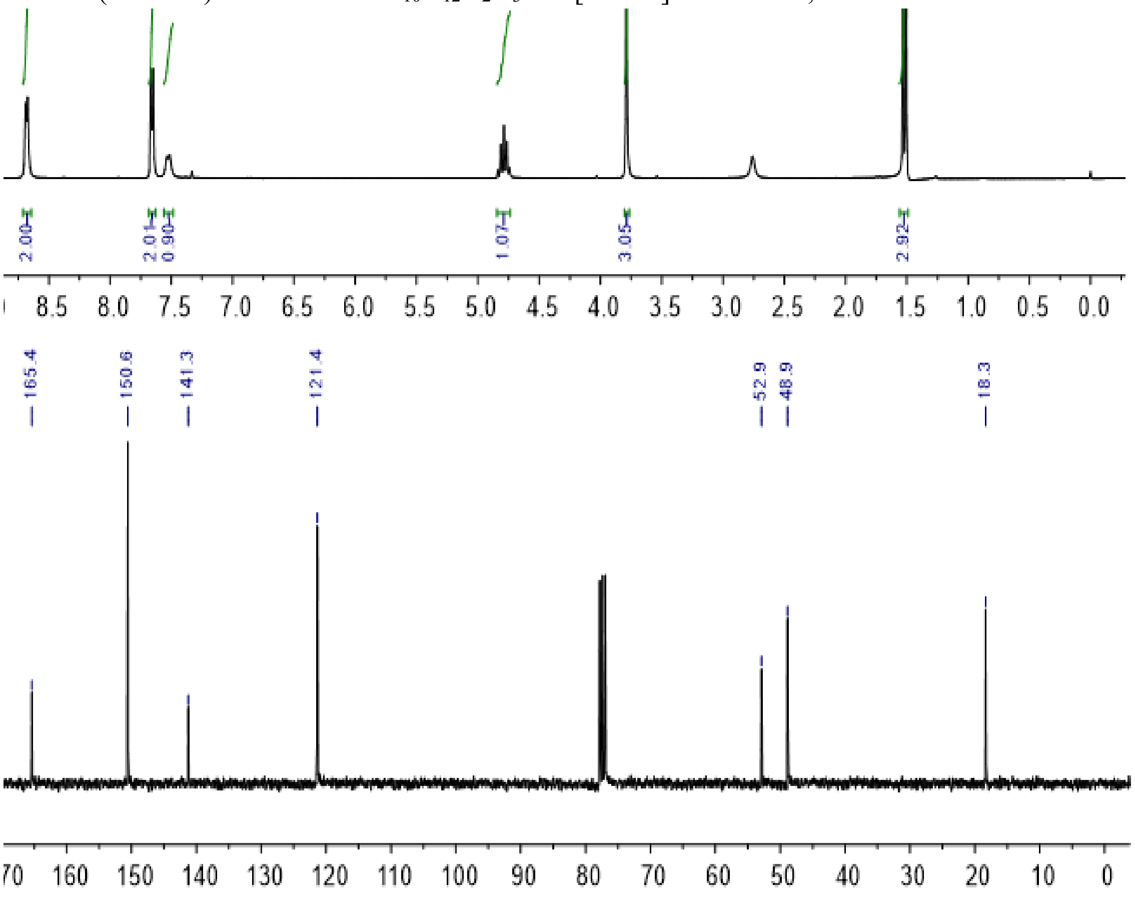

Supplementary Figure 3. ^{1}H and ^{13}C NMR spectra of ligand **2** ($CDCl_3$, 400 and 101 MHz respectively).

4-((trimethylsilyl) ethynyl)pyridine [Ligand 3]

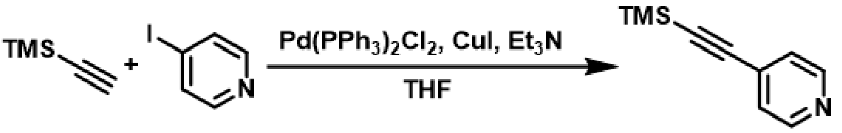

4-Iodopyridine (500 mg, 1.00 equiv.), bis(triphenylphosphine)palladium (II) dichloride ($Pd(PPh_3)_2Cl_2$, 6.85 mg, 0.04 equiv.), triphenylphosphine (10.2 mg, 0.016 equiv.) and copper iodide (CuI, 11.1 mg, 0.024 equiv.) were dissolved in dry THF (20 mL). Next, triethylamine (Et_3N, 10 mL) and ethynyltrimethylsilane (359 mg, 1.5 equiv.) were added and the mixture was stirred for two hours. After removing the solvent, the crude product was chromatographed on silica gel with pentane/ethyl acetate (80:20) as eluent obtaining, after solvent removal, an oil (368 g, 86 % of yield).

The synthetic procedure was previously reported in supplementary reference 1.

^{1}H NMR (400 MHz, $CDCl_3$) δ_H (ppm): 0 (s, 9H), 7.01 (d, 2H), 8.28 (d, 2H).

^{13}C NMR (101 MHz, $CDCl_3$) δ_C (ppm): 0, 100.1, 102.4, 126, 131.4, 150.0.

HRMS (GC-EI-MS) m/z calcd for $C_{10}H_{13}NSi$ $[M+H]^+$: 175.31, found: 175.13.

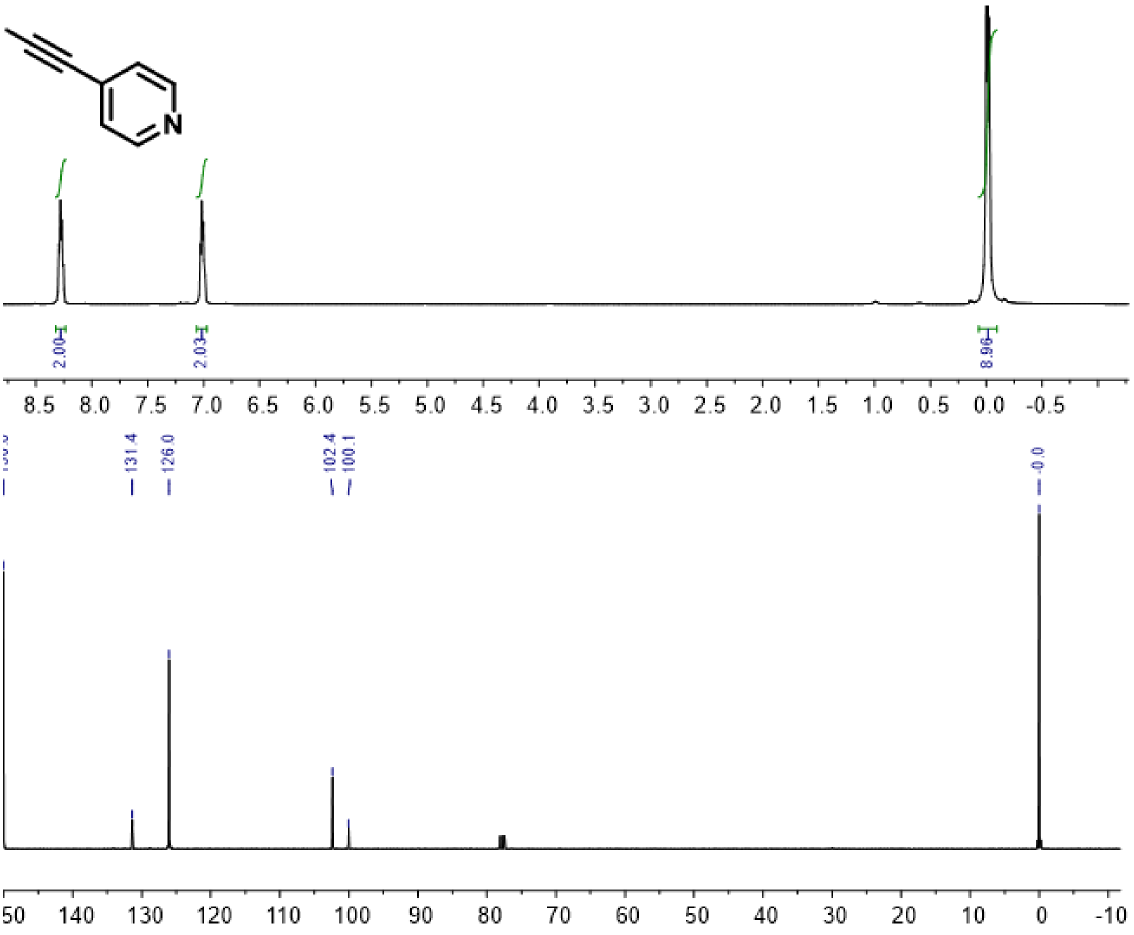

Supplementary Figure 4. ^{1}H and ^{13}C NMR spectra of ligand **3** ($CDCl_3$, 400 and 101 MHz respectively).

General protocol for complexation and deprotection of monomers

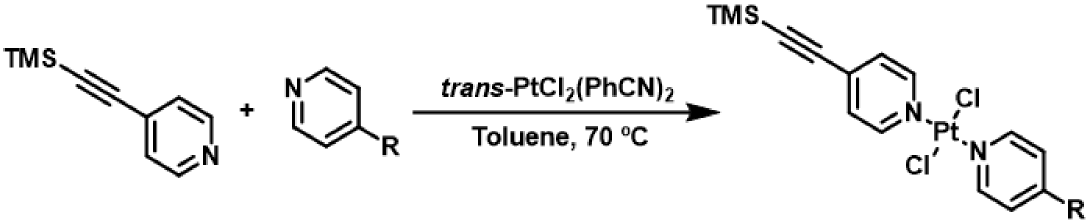

Ligand **3** (1.7 equiv.), *trans*-$PtCl_2(PhCN)_2$ (1.35 equiv.) and the corresponding ligand **1** or **2** (1 equiv.) were placed in a pressure tube and subjected to five vacuum/Ar cycles. Subsequently, dry and degassed toluene was added and the mixture was heated to 70 °C and stirred at this temperature for one week. After evaporating the solvent, the crude was chromatographed on silica gel with pentane/ethyl acetate (80:20) as eluent.

**Ligands**	**Ligand 4 (mg)**	**Ligand X (mg)**	***trans*-PtCl$_2$(PhCN)$_2$ (mg)**	**Toluene (mL)**	**Yield (%)**
**3** and **1**	126.1	100	269.8	4	38.1
**3** and **2**	171.7	120	367.4	4	36.1

Supplementary Table 1. Complexation conditions of the different ligands (**X** = **1** or **2** for ligand-**1** and ligand-**2** respectively).

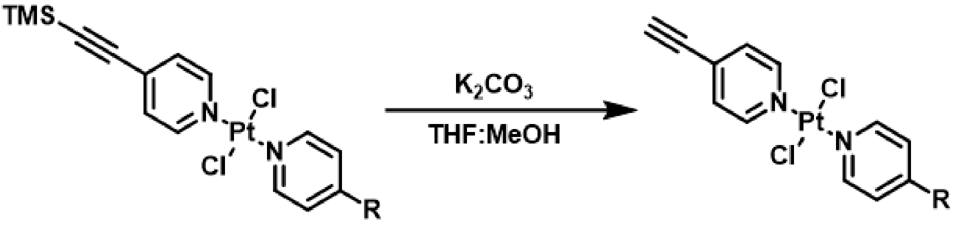

The corresponding Pt(II) complexes with the protected alkyne was dissolved in a mixture of THF:MeOH (3:1). Next K_2CO_3 was added (1.20 equiv.) and the reaction was stirred at r.t. for 1 hour. The crude was chromatographed on silica gel with pentane/ethyl acetate (60:40) as eluent.

	**TMS-mono-(*S*)-X (mg)**	**K$_2$CO$_3$ (mg)**	**THF (mL)**	**MeOH (mL)**	**Yield (%)**
**mono-(*S*)-1**	164	40.1	9	3	95.1
**mono-(*S*)-2**	130	33.2	9	3	96.2

Supplementary Table 2. Deprotection conditions of the different ligands (**X** = **1** or **2** for TMS-mono-(*S*)-**1** and TMS-mono-(*S*)-**2** respectively).

mono-(*S*)-1

^{1}H NMR (400 MHz, $CDCl_3$) δ_H (ppm): 0.98 (t, 3H), 2.28 (m, 1H), 3.53 (s, 1H), 3.79 (s, 1H), 4.71 (dd, 1H), 6.81 (d, 1H), 7.34 (d, 2H), 7.65 (d, 2H), 8.88 (d, 2H), 9.04 (d, 2H).

^{13}C NMR (101 MHz, $CDCl_3$) δ_c (ppm): 18.0, 19.0, 31.5, 52.6, 57.9, 79.4, 86.2, 122.8, 127.7, 133.2, 142.9, 153.4, 154.4, 163.4, 172.0.

HRMS (ESI-MS) m/z calcd for $C_{19}H_{21}Cl_2N_3O_3PtNa^+$ $[M+Na]^+$: 627.0500, found: 627.0508.

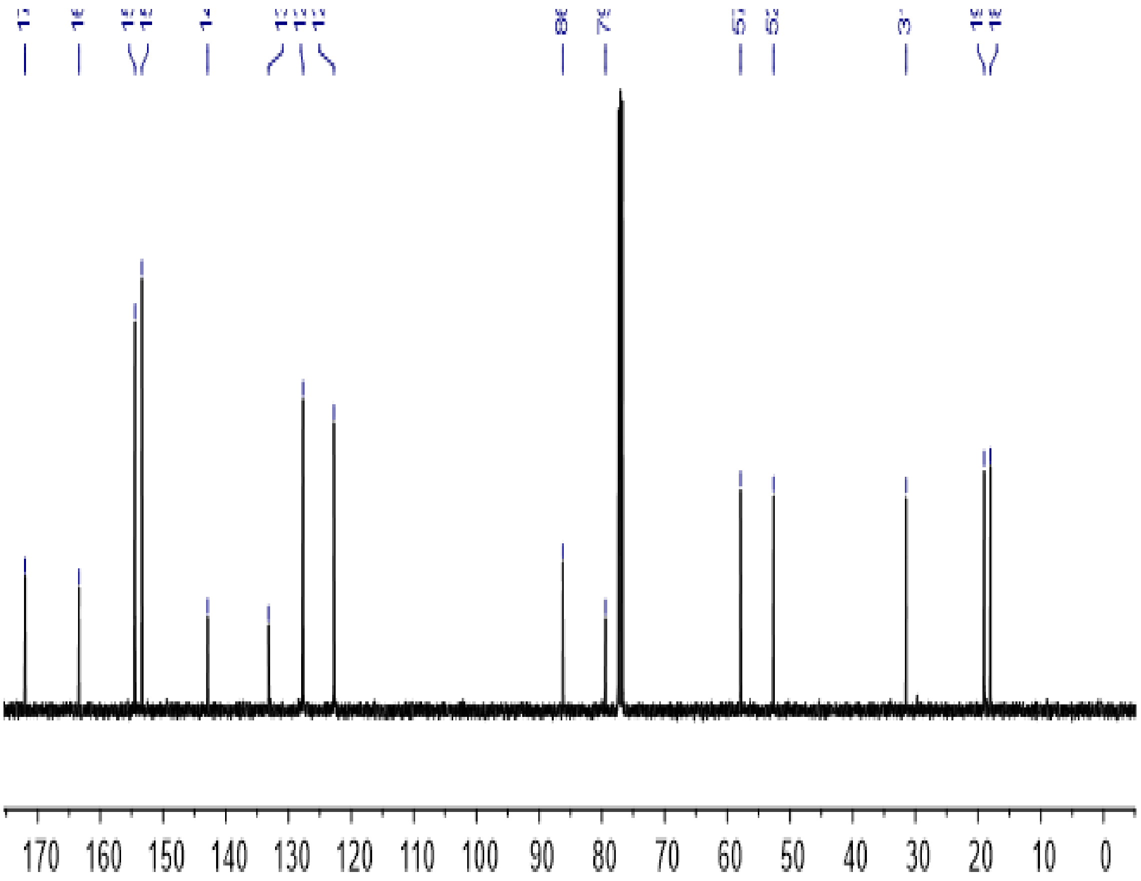

Supplementary Figure 5. ^{1}H and ^{13}C NMR spectra of mono-(*S*)-**1** ($CDCl_3$, 400 and 101 MHz respectively).

mono-(*S*)-2

^{1}H NMR (400 MHz, $CDCl_3$) δ_H (ppm): 1.51 (d, 3H), 3.53 (s, 1H), 3.80 (s, 3H), 4.73 (quint, 1H), 7.10 (d, 1H), 7.34 (d, 2H), 7.63 (d, 2H), 8.87 (d, 2H), 9.02 (d, 2H).

^{13}C NMR (101 MHz, $CDCl_3$) δ_c (ppm): 18.1, 48.9, 52.9, 79.4, 86.3, 122.8, 127.7, 133.2, 142.6, 153.4, 154.4, 162.9, 173.0.

HRMS (ESI-MS) m/z calcd for $C_{17}H_{17}Cl_2N_3O_3PtNa^+$ $[M+Na]^+$: 600.3142, found: 600.0172.

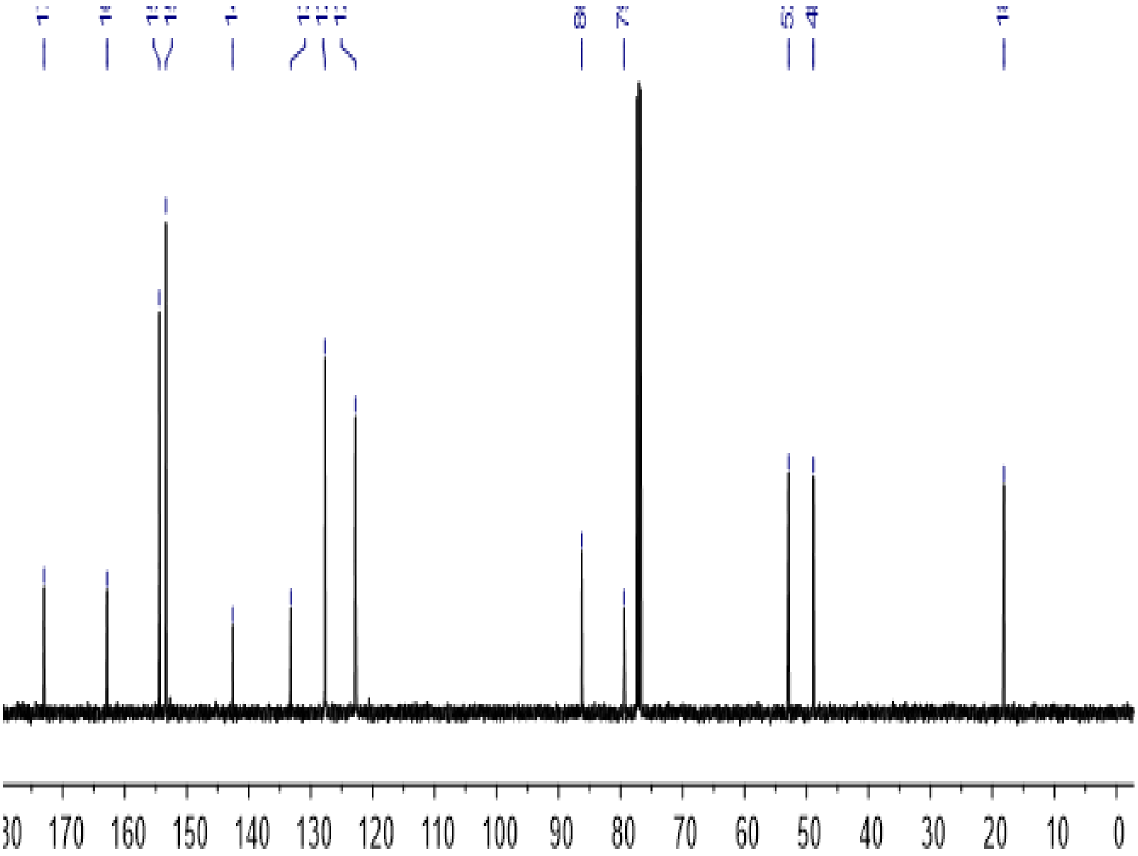

Supplementary Figure 6. ^{1}H and ^{13}C NMR spectra of mono-(*S*)-**2** ($CDCl_3$, 400 and 101 MHz respectively).

Synthesis of Polymers

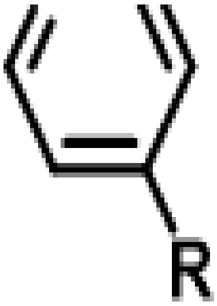

The reaction flask (sealed ampoule) was dried under vacuum and argon flushed for three times before monomer was added as a solid. Dry THF was added with a syringe. Next, a solution of rhodium norbornadiene chloride dimer, $[Rh(nbd)Cl]_2$, and ET_3N in dry THF was added to the reaction that is under stirring at 30 °C. After 6 hours, the resulting polymer was diluted in CH_2Cl_2 and it was precipitated in methanol and centrifuged (2 times), reprecipitated in hexane and centrifuged again.

**Ligands**	**mono-(*S*)-X (mg)**	**THF (mL)**	**[Rh(nbd)Cl]2 (mg)**	**ET_3N (µL)**	**Yield (%)**
**poly-(*S*)-1**	75	0.25	1.2	5.0	95.1
**poly-(*S*)-2**	75	0.26	1.2	5	96.2

Supplementary Table 3. Polymerization conditions of the different polymers (**X** = **1** or **2** for mono-(*S*)-**1** and mono-(*S*)-**2** respectively).

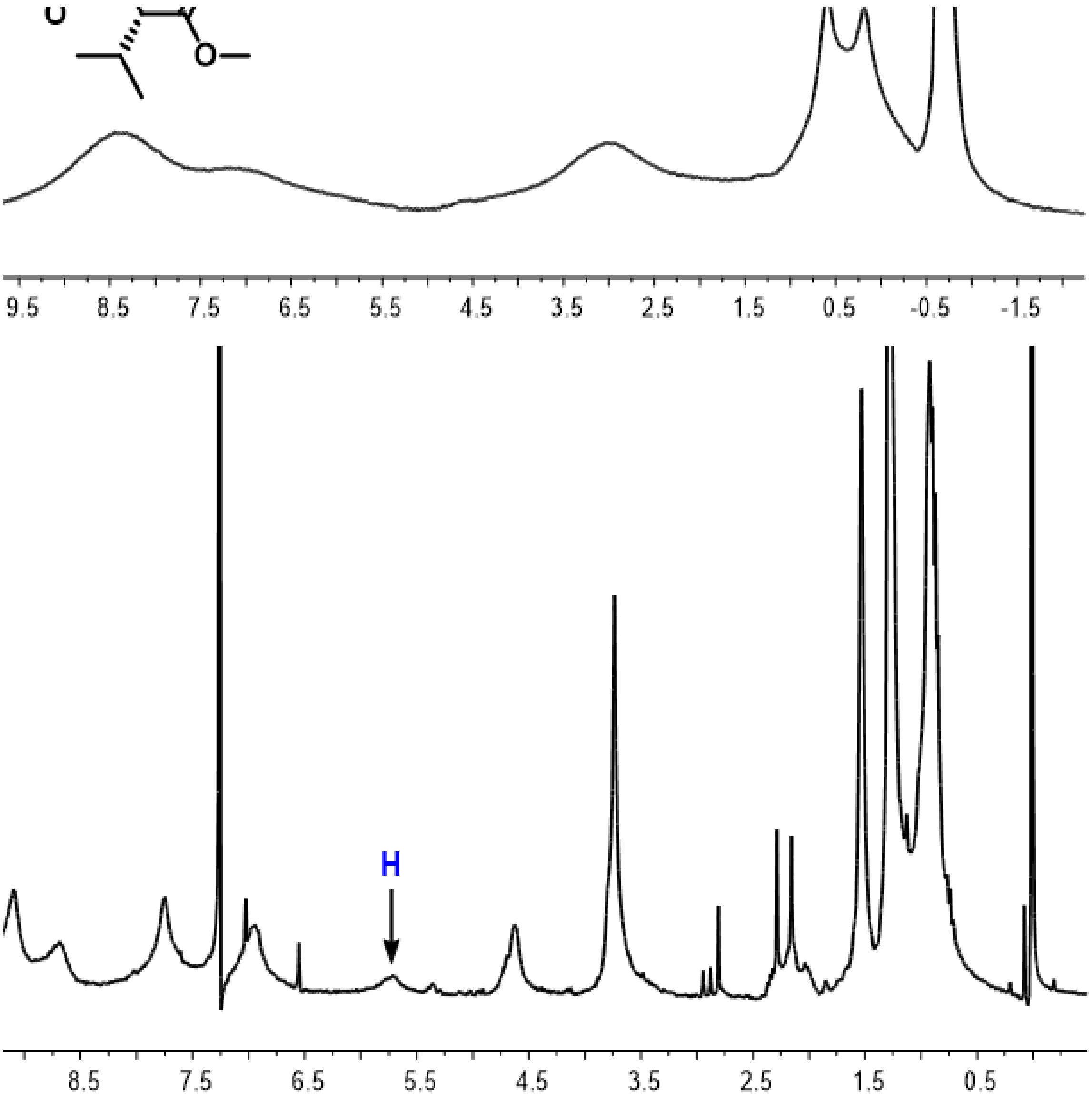

Supplementary Figure 7. ^{1}H NMR spectra of poly-(*S*)-**1** as a) solid sample, ^{1}H spectra NEO-750 MHz with a 1.3 mm rotor and b) in $CDCl_3$, 400 MHz.

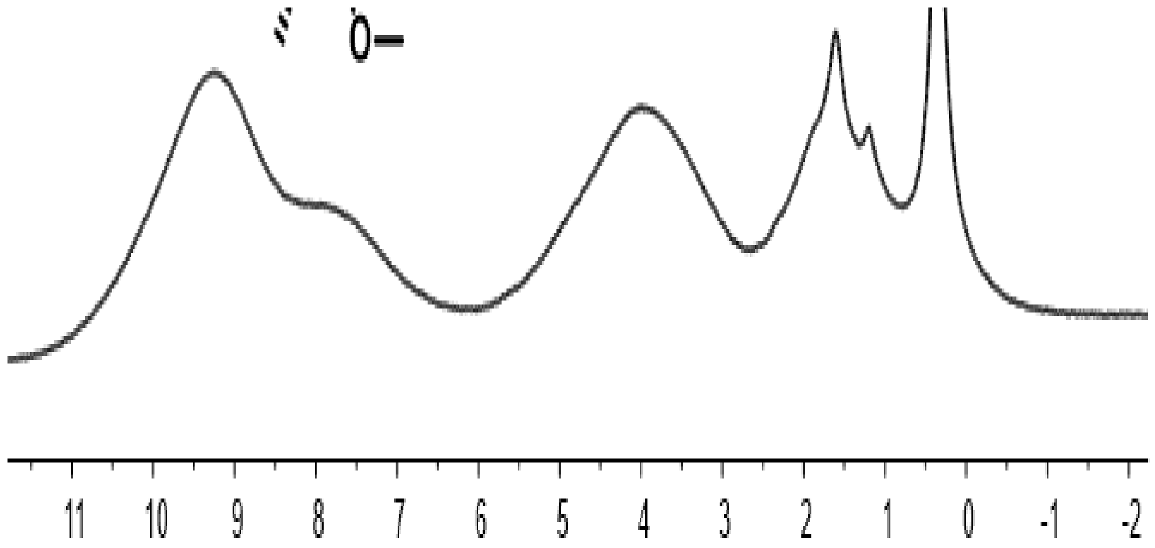

Supplementary Figure 8. ^{1}H spectra of poly-(*S*)-**2** (Solid sample, NEO-750 MHz with a 1.3 mm rotor).

Elemental Analysis

A solid sample (5 mg) of the corresponding monomer was kept under vacuum for 24 hours before being submitted to elemental analysis.

	**C (%)**<br>**Estimated/measured**	**H (%)**<br>**Estimated/measured**	**N (%)**<br>**Estimated/measured**
**mono-(*S*)-1**	37.70/38.11	3.50/3.72	6.94/6.69
**mono-(*S*)-2**	35.37/36.78	2.97/3.49	7.28/6.69

Supplementary Table 4. Tabulated results of elemental analysis for the different monomers analysed.

Spectroscopy Studies

Raman

Samples were prepared by drop casting 0.5 mg/mL solutions of the corresponding polymer over a microscope slide. After solvent evaporation, the spectra were recorded on a Renishaw confocal Raman spectrometer. For DMF solutions, samples were deposited on the microscope slide at 175 °C.

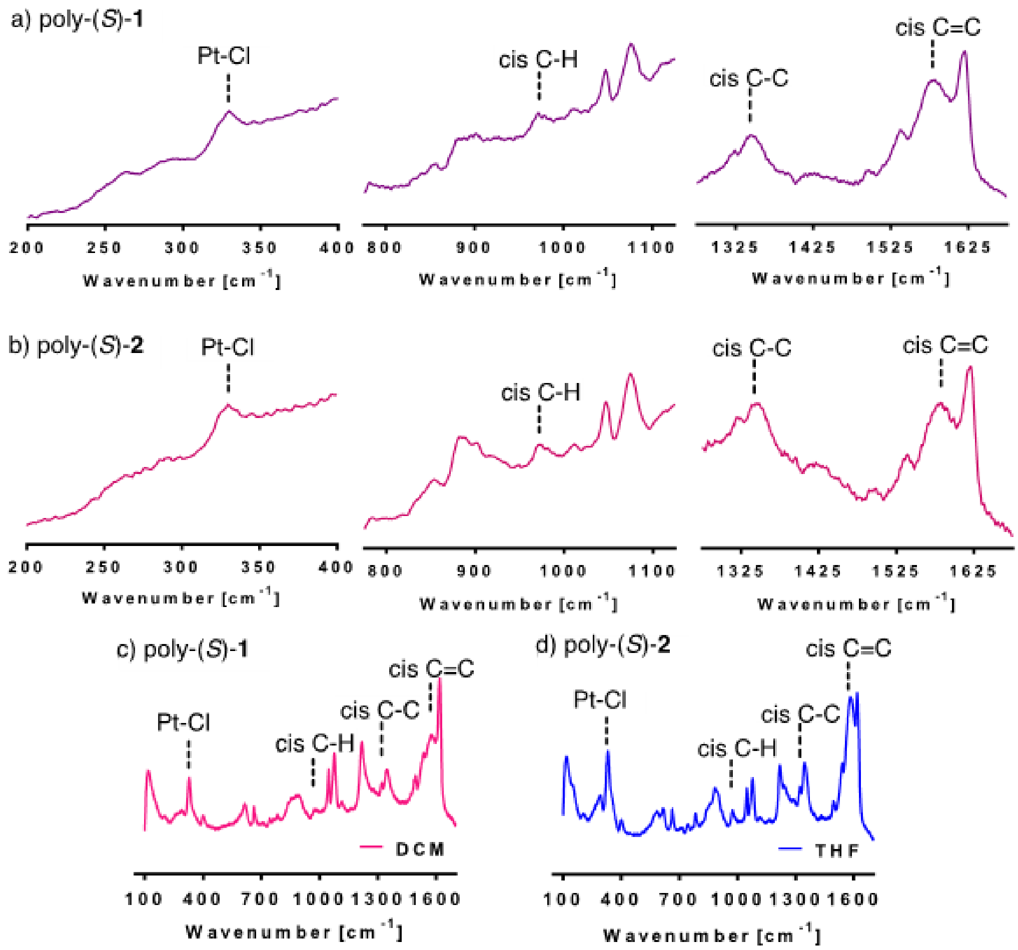

Supplementary Figure 9. Raman spectra in DMF of a) poly-(*S*)-**1** and b) poly-(*S*)-**2**. Raman spectra of poly-(*S*)-**1** in c) DCM and d) THF.

ATR/FT-IR

Solid IR spectra were recovered to confirm the polymerization of the monomers as result of the dissaperence of the C≡C vibration around 2150 cm^{-1}.

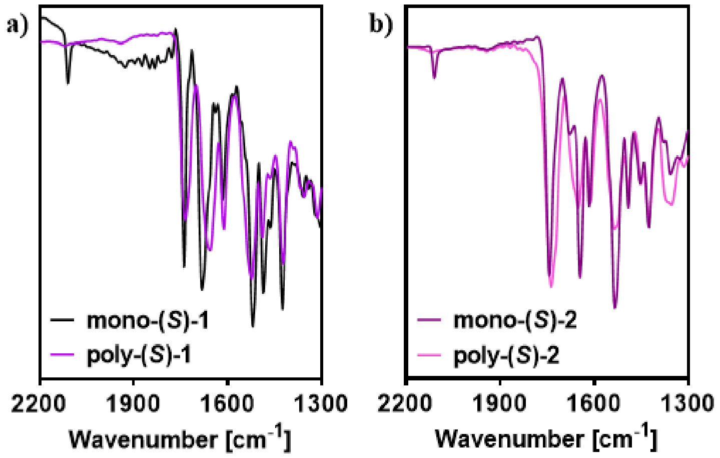

Supplementary Figure 10. ATR/FT-IR spectra of a) mono-(*S*)-**1** and poly-(*S*)-**1** and b) mono-(*S*)-**2** and poly-(*S*)-**2**.

Thermal Studies

DSC studies

A polymer sample, pre-dried, was introduced in an aluminum pan and heated from 60 °C to 350 °C with a heating rate of 5 °C/min. To compare all polymers at the same conditions, DSC thermograms were recorded immediately after polymerization (see section synthesis of polymers for details). Thus, the results for poly-(*S*)-**1** and poly-(*S*)-**2** show thermograms of the polymers with a clear transition for the *c-c* to *t-t* transformation. However, despite the stiffness generated by the bispyridyldichlorido platinum(II) complexes, the *c-t* to *c-c* transition is weak. For poly-(*S*)-**1**, the thermogram was also obtained after dissolving in DCM and evaporating the solvent. This thermogram was compared with the one obtained in DMF, due to the formation of a *c-c* scaffold in this solvent, the peak associated to *the c-t* to *c-c* transition disappears. Also, a new and broad exothermic peak appears between 120-260 °C, probably as result of the strong supramolecular assembly between the bispyridyldichlorido platinum(II) groups.

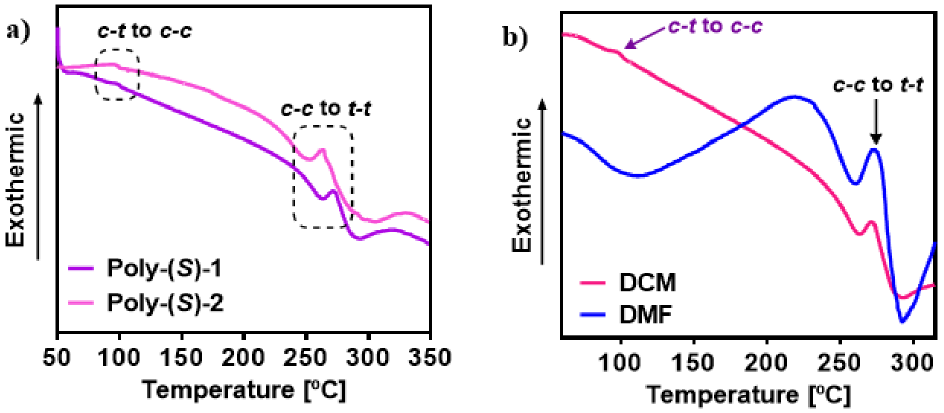

Supplementary Figure 11. DSC thermograms of a) the three polymers after the polymerization and b) poly-(*S*)-**1** in DCM and DMF scaffolds.

TGA studies

Solid polymer samples were kept under vacuum for 12 hours. After this time, the samples were introduced in a platinum pan and heated from 40 °C to 800 °C with a heating rate of 10 °C/min.

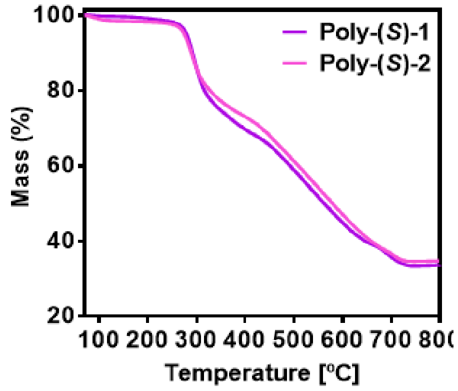

Supplementary Figure 12. TGA thermograms of a) poly-(*S*)-**1** and b) poly-(*S*)-**2**.

VT-CD Experiments

Variable temperature circular dichroism (VT-CD) experiments were measured in a 1 mm quartz cell on a Jasco-1500 with a sample concentration of 0.6 mg/mL.

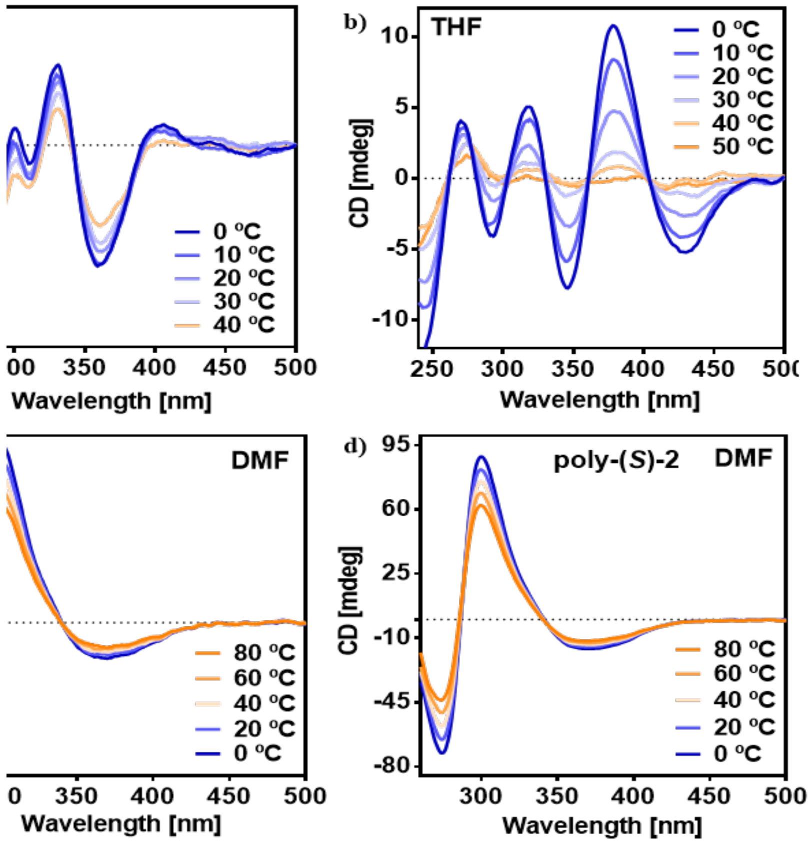

Supplementary Figure 13. VT-CD spectra of poly-(*S*)-**1** in a) DCM, b) THF, c) DMF and d) poly-(*S*)-**2** in DMF. [poly-(*S*)-**1**]= 0.6 mg/mL, [poly-(*S*)-**2**]= 0.6 mg/mL.

Photoluminescence properties

Photoluminescence properties were studied on a Horiba Fluoromax-Plus-C fluorimeter using a 10 mm quartz cuvette and with a sample concentration of 0.6 mg/mL. No emission was observed in poly-(*S*)-**1** studies.

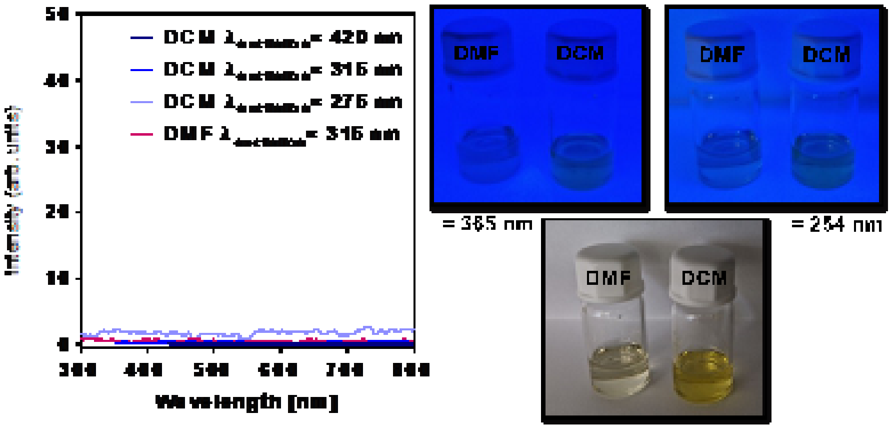

Supplementary Figure 14. Experimental fluorescence spectra of poly-(*S*)-**1** in DMF and DCM. [poly-(*S*)-**1**]= 0.6 mg/mL.

IR analysis of poly-(*S*)-1

IR spectra of poly-(S)-**1** were obtained on a JASCO-FTIR-6800 equipped with a CaF2 cell with a path length of 0.1 mm.

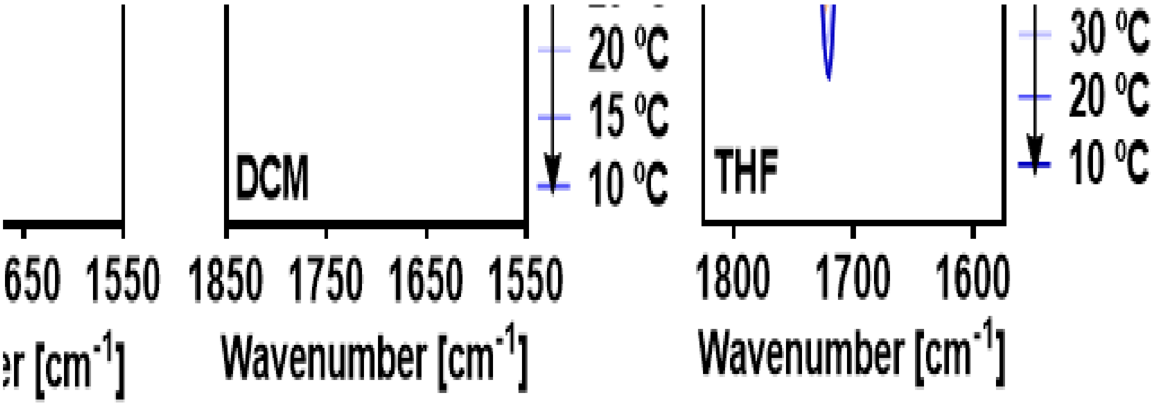

Supplementary Figure 15. IR spectra in solution of poly-(*S*)-**1** in a) DCM and THF. VT-IR spectra in solution of poly-(*S*)-**1** in b) DCM and c) THF. [poly-(*S*)-**1**]= 1.0 mg/mL.

Linear Dichroism Experiments

Linear dichroisms (LD) experiments were measured in a 1 mm quartz cell on a Jasco-1500 with a sample concentration of 0.6 mg/mL.

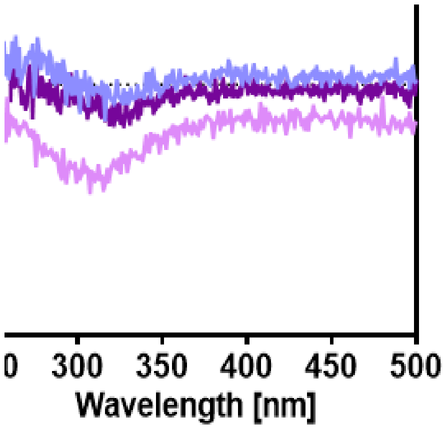

Supplementary Figure 16. LD spectra of poly-(*S*)-**1** in THF, DCM and DMF. [poly-(*S*)-**1**]= 0.6 mg/mL, [poly-(*S*)-**2**]= 0.6 mg/mL.

Spatial distribution of bispyridyldichlorido platinum(II) groups in a *cis-cisoidal/cis-transoidal* helix

Before submitting theoretical calculations, we built 3D models of the bispyridyldichlorido platinum(II) complexes in a *cis-cisoidal* and a *cis-transoidal* helix. Thus, we can observe that the orientation of the chlorido ligands and even the ω_1 angle (relative orientation of the bispyridyldichlorido platinum(II) towards the polyene) is influenced by the steric hindrance generated between upper and lower groups in the helix. As a result, the sign of these angles must reverse with the inversion of the helical sense of the helix. The resulting 3D model submitted for theoretical calculations agrees with these assumptions and the resulted conformations and ECD/UV-vis calculated spectra confirm this idea.

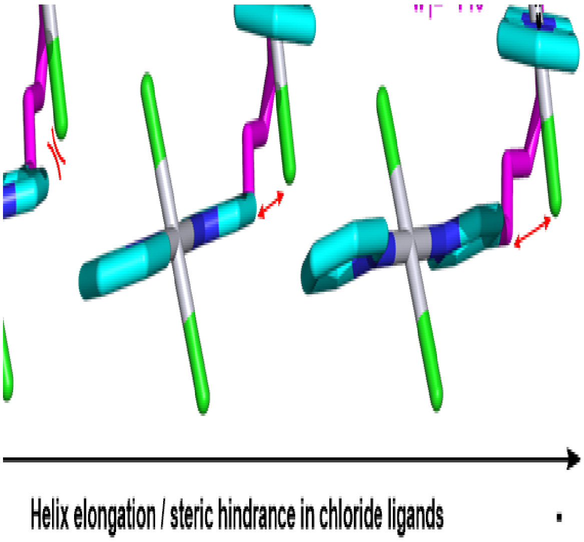

Supplementary Figure 17. 3D model representing the spatial distribution of bispyridyldichlorido platinum(II) groups in a a) *cis-cisoidal* helix and b) *cis-transoidal* helix.

UV-Vis Monomer calculations

Computational studies were carried out with the software package Gaussian 16, rev C.01 (supplementary reference S1). Thus, TD-DFT(wB97XD)/ LANL2DZ) and TD-DFT(rCAM-B3LYP)/ LANL2DZ (supplementary references S2-S5), were done to calculate the theoretical UV-Vis spectrum of mono-(S)-**1**. Both spectra show a good match with the experimental one.

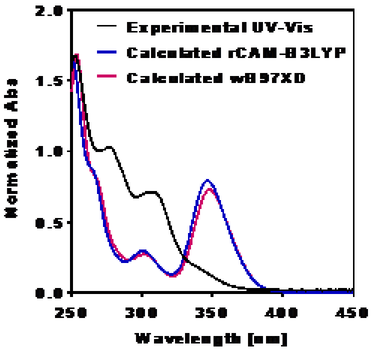

Supplementary Figure 18. Experimental and theoretical UV-vis spectra of mono-(*S*)-**1**.

Calculated UV-Vis spectra, after geometry optimization (see section "computational details"), allow to identify the UV-vis transitions. Thus, the UV-band at higher wavelength —ca. 345 nm— corresponds to MLCT associated with the core of the bispyridyldichlorido Pt^{II} complexes (S_0 to S_9 and S_0 to S_{11}), the band at ca. 305 nm corresponds to *para*-ethynylpyridyl complexed to the dichlorido Pt^{II} (S_0 to S_{26}), while the band at 280 nm is attributed to the para-pyridylbenzamide of (*L*)-Valine-methyl ester complexed to the dichlorido Pt^{II} (S_0 to S_{44}). Finally, the band at 250 nm is related to the benzamide group of the chiral moiety (mainly S_0 to S_{65}).

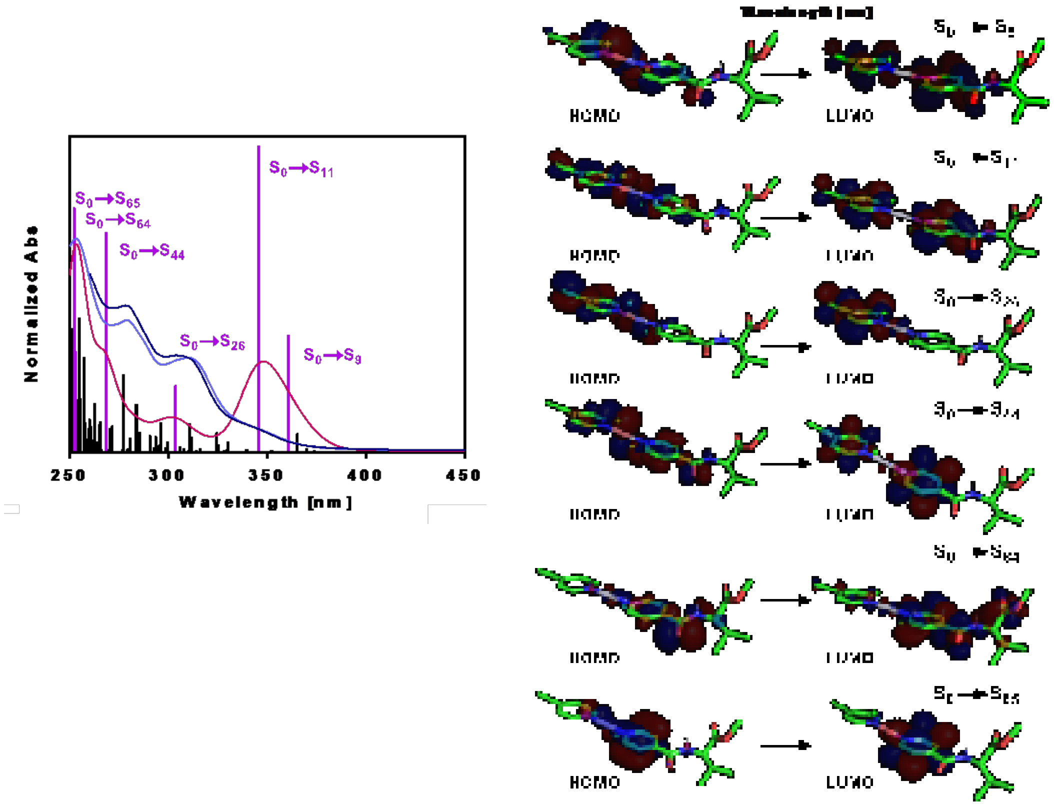

Supplementary Figure 19. Main HOMO and LUMO of the transition assigned to each calculated natural transition orbital (NTO).

Computational Details

Considering the difficulties to carry out theoretical calculations on large polymers, we applied the methodology reported in supplementary reference S3. Thus, oligomers constituted by 9 monomer repeating units were built using Spartan 18 (supplementary reference S4). The generated structure was adjusted according to the information obtained from several techniques such as Raman, IR, CD, UV-vis and the geometry calculated for the monomeric stacks in a left-handed helix. Finally, the time-dependent density functional theory (TD-DFT, supplementary reference S5) was used together with the rCAM-B3LYP density functional (supplementary reference S6) and the LANL2DZ basis set (supplementary reference S7). We included 180 excitation energies in the calculation for the scaffold. The resulting ECD spectrums were selected with a full width at half height (FWHM) of 0.33 eV. Moreover, we evaluated a correction factor for lambda as the difference between the theoretical and experimental wavelengths, and we shifted the rest of the theoretical spectra accordingly. In a similar way, the intensity was rescaled for the theoretical values.

For monomer calculations, individual monomer units were optimized in both geometries (to achieve the final left and right-handed orientation) using the density functional theory (DFT) together with the rCAM-B3LYP functional and the LANL2DZ basis set. The resulting geometries were used to build and stack three monomeric units, which were optimized again using the same parameters. Next, theoretical ECD spectra for both structures were calculated using the time-dependent density functional theory (TD-DFT), applying the rCAM-B3LYP density functional theory and the LANL2DZ basis set. The resulting ECD spectrum was selected with a full width at half height (FWHM) of 0.33 eV. Moreover, the theoretical UV-vis spectrum of the monomer was calculated from the previously optimized geometry for one monomeric unit using the time-dependent density functional theory (TD-DFT) together the wB97XD density functional and the LANL2DZ basis set. The resulting UV-vis spectrum was selected with a full width at half height (FWHM) of 0.2 eV. Also, we evaluated a correction factor for lambda as the difference between the theoretical and experimental wavelengths, and we shifted the rest of the theoretical spectrum accordingly. This correction factor was also applied to the ECD calculated of the monomeric stacks.

Polymerization degree effects

To determine the role of the polymerization degree in the chiroptical properties of poly-(S)-**1**, an oligomer of poly-(*S*)-**1** was prepared according to Prof. Maeda´s protocol (supplementary reference S8).

GPC studies show the presence of two different peaks at 19 and 26 min, which correspond to a polymer (DP = 247) and an oligomer (DP = 24) of poly-(S)-**1** respectively. ECD studies show in both cases the same ECD trace, although more intense for the polymer (g_{abs} (poly-(*S*)-**1** = 1.05 x 10^{-3}; oligomer-(*S*)-**1** = 6.87 x 10^{-4}). This fact indicates that in large polymers, the relationship between monomer repeating units at internal positions vs. the helix edge is larger than in the oligomer. As a result, there are more bispyridyldichlorido platinum(II) intrapendant supramolecular interactions, that result in a large ECD spectrum.

a) b)

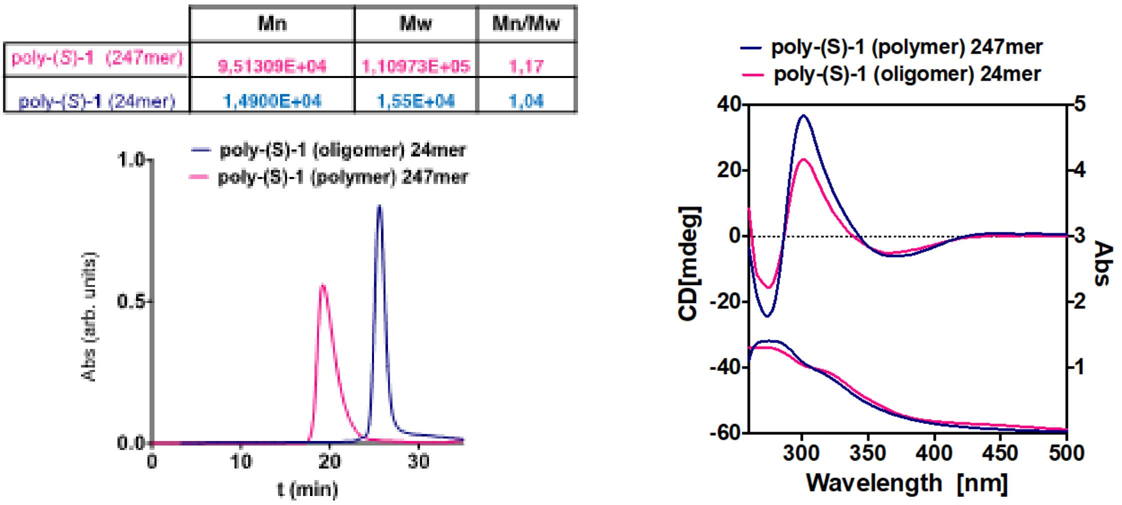

	Mn	Mw	Mn/Mw
poly-(*S*)-1 (247mer)	9,51309E+04	1,10973E+05	1,17
poly-(*S*)-1 (24mer)	1,4900E+04	1,55E+04	1,04

Supplementary Figure 20. a) GPC traces and (b) spectroscopic studies (ECD, UV-vis) of an oligomeric and polymeric version of poly-(S)-**1**. [poly-(*S*)-**1**]= 0.6 mg/mL DMF.

Supplementary References

1. R. Ziessel, J. Suffert, M. T. Youinou, *J. Org. Chem.*, **1996**, *61*, 6535-6546.

2. Gaussian 16, Revision C.01, M. J. Frisch, G. W. Trucks, H. B. Schlegel, G. E. Scuseria, M. A. Robb, J. R. Cheeseman, G. Scalmani, V. Barone, G. A. Petersson, H. Nakatsuji, X. Li, M. Caricato, A. V. Marenich, J. Bloino, B. G. Janesko, R. Gomperts, B. Mennucci, H. P. Hratchian, J. V. Ortiz, A. F. Izmaylov, J. L. Sonnenberg, D. Williams-Young, F. Ding, F. Lipparini, F. Egidi, J. Goings, B. Peng, A. Petrone, T. Henderson, D. Ranasinghe, V. G. Zakrzewski, J. Gao, N. Rega, G. Zheng, W. Liang, M. Hada, M. Ehara, K. Toyota, R. Fukuda, J. Hasegawa, M. Ishida, T. Nakajima, Y. Honda, O. Kitao, H. Nakai, T. Vreven, K. Throssell, J. A. Montgomery, Jr., J. E. Peralta, F. Ogliaro, M. J. Bearpark, J. J. Heyd, E. N. Brothers, K. N. Kudin, V. N. Staroverov, T. A. Keith, R. Kobayashi, J. Normand, K. Raghavachari, A. P. Rendell, J. C. Burant, S. S. Iyengar, J. Tomasi, M. Cossi, J. M. Millam, M. Klene, C. Adamo, R. Cammi, J. W. Ochterski, R. L. Martin, K. Morokuma, O. Farkas, J. B. Foresman, and D. J. Fox, Gaussian, Inc., Wallingford CT, **2016**.

3. B. Fernández, R. Rodríguez, A. Rizzo, E. Quiñoá, R. Riguera, F. Freire, *Angew. Chem. Int. Ed.* **2018**, *57*, 3666-3670.

4. Y. Shao, L.F. Molnar, Y. Jung, J. Kussmann, C. Ochsenfeld, S.T. Brown, A.T.B. Gilbert, L.V. Slipchenko, S.V. Levchenko, D.P. O'Neill, R.A. DiStasio Jr., R.C. Lochan, T. Wang, G.J.O. Beran, N.A. Besley, J.M. Herbert, C.Y. Lin, T. Van Voorhis, S.H. Chien, A. Sodt, R.P. Steele, V.A. Rassolov, P.E. Maslen, P.P. Korambath, R.D. Adamson, B. Austin, J. Baker, E.F.C. Byrd, H. Dachsel, R.J. Doerksen, A. Dreuw, B.D. Dunietz, A.D. Dutoi, T.R. Furlani, S.R. Gwaltney, A. Heyden, S. Hirata, C-P. Hsu, G. Kedziora, R.Z. Khalliulin, P. Klunzinger, A.M. Lee, M.S. Lee, W.Z. Liang, I. Lotan, N. Nair, B. Peters, E.I. Proynov, P.A. Pieniazek, Y.M. Rhee, J. Ritchie, E. Rosta, C.D. Sherrill, A.C. Simmonett, J.E. Subotnik, H.L. Woodcock III, W. Zhang, A.T. Bell, A.K. Chakraborty, D.M. Chipman, F.J. Keil, A.Warshel, W.J. Hehre, H.F. Schaefer, J. Kong, A.I. Krylov, P.M.W. Gill and M. Head-Gordon, *Phys. Chem. Chem. Phys*., **2006**, *8*, 3172

5. E. Runge, E. K. U. Gross, *Phys. Rev. Lett.* **1984**, *52*, 997-1000.

6. A. J. Cohen, P. Mori-Sánchez, W. Yang, *J. Chem. Phys.* **2007**, *126*, 191109.

7. P. J. Hay, W. R. Wadt, *J. Chem. Phys.*, **1985**, *82*, 270-283.

8. T. Taniguchi, T. Yoshida, K. Echizen, K. Takayama, T. Nishimura, K. Maeda, *Angew. Chem. Int. Ed.* **2020**, *59*, 8670-3680.
